# Cross-sectional validity of the EQ-5D-Y as a generic health outcome instrument in children and adolescents with cystic fibrosis in Germany

**DOI:** 10.1186/1471-2431-9-55

**Published:** 2009-08-28

**Authors:** Daniela Eidt-Koch, Thomas Mittendorf, Wolfgang Greiner

**Affiliations:** 1 Centre for Health Economics and Health System Research, Leibniz University Hannover, Germany; 2 Health Economics and Health Care Management, University of Bielefeld, Germany; 3 herescon gmbh, Hannover, Germany

## Abstract

**Background:**

Quality of life is recognized as an important additional outcome measure in clinical trials and health economic evaluations. The EQ-5D is an important generic health outcome instrument often used for economic evaluations as a complement with disease-specific outcome measures. In this study quality of life data was assessed using the EQ-5D-Y (new EQ-5D version for children and adolescents) and the Cystic Fibrosis Questionnaire (CFQ). The objective of the study is to evaluate the cross-sectional validity of the EQ-5D-Y as a generic health outcome instrument in children and adolescents with cystic fibrosis in Germany.

**Methods:**

In 2006 a multi-centre study was conducted in four cystic fibrosis centres in Germany. Quality of life data from 96 patients between eight and seventeen years was collected using the EQ-5D-Y as a generic outcome instrument and the Cystic Fibrosis Questionnaire (CFQ) as a disease-specific instrument. Results of both instruments were compared by statistical analyses using Spearman's rank correlations.

**Results:**

44.6% of the patients stated that they had no problems in any of the EQ-5D-Y dimensions. Several low to high correlations between separate dimensions and the visual analogue scale of the EQ-5D-Y and the different scales of the CFQ for children, their parents and adolescents can be presented in this paper. Looking at the five EQ-5D-Y dimensions the highest correlation (r_S _= 0.625, p = 0.01) was found between the dimension 'mobility' and the CFQ scale 'physical functioning' in adolescent patients. The overall highest correlation was found between the 'subjective health perception' and the visual analogue scale (r_S _= 0.744, p = 0.01) in adolescent patients older than 13 years.

**Conclusion:**

The EQ-5D-Y can be considered a cross-sectional valid generic health outcome instrument which reflects differences in health according to the progression of the life-long chronic disease cystic fibrosis.

## Background

Quality of life is recognized as an important additional outcome measure in clinical trials and health economic evaluations. In the past two decades many instruments for the assessment of quality of life have been developed. Most of the developed instruments for the assessment of quality of life are for adults. However, quality of life instruments for children, which are applicable in healthy as well as chronically ill children, are important and their use in clinical research should be expanded [1].

The EQ-5D is an important generic health outcome instrument often used for economic evaluations as a complement with disease-specific outcome measures. As of now, the EQ-5D is only validated for adults. Therefore the internationally applicable child friendly version EQ-5D-Y has been established by the EuroQol group and translated into many different languages (Wille N, the EuroQol Youth Task Force & Ravens-Sieberer U: Development of a child-friendly EQ-5D: the EQ-5D-Y international version, submitted). In this multi centre study quality of life data from cystic fibrosis children and adolescents was assessed using the EQ-5D-Y as a generic health outcome instrument and the Cystic Fibrosis Questionnaire (CFQ) as a disease-specific instrument in cystic fibrosis patients.

Cystic fibrosis is a complex lifelong chronic disease caused by genetic mutations. In most cases multiple organ systems are affected, most patients especially suffer from pancreatic insufficiency and lung function problems. Hence, this chronic condition leads to irreversible organ damages with patients having a life expectancy of only 29 years in 2007 [2].

In this article the results of the EQ-5D-Y as well as the results of the CFQ will be analysed and correlations between dimensions and scales of the instruments will be demonstrated. The aim is to assess the cross-sectional validity of the EQ-5D-Y.

## Methods

The multi-centre study was based on single cross-sectional data collection from children and adolescents between 8 to 17 years with cystic fibrosis in continuous ambulatory treatment in four German cystic fibrosis centres (Hannover Medical School, Johann Wolfgang Goethe University Hospital, Children's Hospital, University of Heidelberg, and the Dr. von Haunersches Kinderspital, Ludwig Maximilian University Munich) between April and August 2006. The study has been approved by the ethic committees of the involved clinics. The patients gave their consent for the study.

Two instruments were used to collect quality of life data: the EQ-5D-Y as a generic health outcome instrument and the CFQ as a disease-specific instrument.

The EQ-5D consists of five dimensions of health: mobility, self-care, usual activities, pain/discomfort and anxiety/depression. The respondent can choose the levels no (1), some (2) or extreme (3) problems. The result is a health profile, e.g. a patient with the health profile 11223 has no problems with mobility and self-care, some problems with everyday activities and pain/discomfort and extreme problems with happiness/worry/sadness, respectively. The visual analogue scale (VAS) included in the EQ-5D is used to capture the patients subjective health perception between worst imaginable health state (score 0) and best imaginable health state (score 100) [3]. The EQ-5D was initially constructed and validated for adult patients. The EuroQol group now created a version for children in different languages. The EQ-5D-Y differs from the adult version in changes of words which especially were adapted for children. The German child-friendly version of the EQ-5D (EQ-5D-Y) is available at the EuroQol group . First studies about the child-friendly EQ-5D were presented on the 23^rd ^Scientific Plenary Meeting of the EuroQol Group in Barcelona, Spain, September 14–16, 2006 [4-10].

The Cystic Fibrosis Questionnaire (CFQ) is a disease-specific instrument for quality of life measurement in cystic fibrosis patients developed in France. Translations and validations for German language are available [11]. The CFQ has been developed in different versions for children from 8 to 13 years (CFQ-k) and their parents (CFQ-e) as well as a version for adolescents and adults aged 14 years or older (CFQ 14+). The CFQ consists of 35 (CFQ-k), 44 (CFQ-e) or 50 (CFQ 14+) items containing different modules (quality of life, general health perception, symptoms), dimensions and scales, respectively. Scales in the generic dimensions of quality of life are physical functioning, energy, emotional state, social limitations, everyday life/school problems, in the disease specific dimensions body image, eating disturbance and treatment burden. Scales for the symptoms are weight problems, respiratory and digestive symptoms. Answers of the patients are transformed into scales between 0 (worst) and 100 (best).

According to similar concepts and content of the questionnaires high correlations (r_S _> 0.5, p < 0.01) are especially hypothesized for example between the EQ-5D-Y dimension 'mobility' and the CFQ dimension 'physical functioning', between the EQ-5D-Y dimension 'happiness/worry/sadness' and the CFQ dimension 'emotional state' as well as between the EQ-5D-Y VAS and the CFQ dimension 'subjective health perception'. Moderate (0.3 ≤ r_S _≤ 0,5) or mild correlations (r_S _< 0.3) are hypothesized for example between the EQ-5D-Y dimension 'pain/discomfort' and the and the CFQ scales 'respiratory symptoms' and 'digestive symptoms' as well as the EQ-5D-Y VAS and the CFQ scales 'physical functioning' and 'emotional state' could be expected for. According to different issues no significant correlations are for example hypothesized between the EQ-5D-Y dimension 'self care' and especially for the CFQ scales 'body image'.

A subgroup analysis was made for patients with no problems on EQ-5D-Y (health profile 11111).

Statistical analyses in this paper were performed using SPSS and EXCEL.

## Results

### Socio-demographic and clinical data

Data from 96 patients with an age between 8 and 17 years were collected. 55 patients (57.3%) were between 8 and 13 years (mean age: 10.8 years) and 41 patients (42.7%) were between 14 and 18 years old (mean age: 15.9 years). Patients in this multi-centre study were treated in the ambulatory cystic fibrosis centres of the Hanover Medical School (22 patients), Johann Wolfgang Goethe University Hospital (24 patients), Children's Hospital, University of Heidelberg (5 patients), and the Dr. von Haunersches Kinderspital, Ludwig Maximilian University Munich (45 patients). Clinical data is shown in table 1.

**Table 1:** Clinical data

**Variable**	**Children** **(8 to 13 years)** **(n = 55)**	**Adolescents** **(14 to 17 years)** **(n = 41)**	**Significance level**
Sex (male)	43.6% (n = 24)	58.5% (n = 24)	

Age (mean/SD)	10.8/1.7	15.9/1.80	

% vital capacity (mean/SD)	92.5%/11.9% (n = 47)	97.2%/13.1% (n = 34)	p = 0.082

% FEV_1 _(mean/SD)	93.6%/15.2% (n = 47)	90.7%/20.3% (n = 34)	p = 0.618

% MEF_25 _(mean/SD)	68.4%/41.7% (n = 47)	58.9%/37.5% (n = 34)	p = 0.273

Bacterial colonization of the lung	63.6% (n = 35)	73.2% (n = 30)	p = 0.009

Pneumothorax	1.8% (n = 1)	0% (n = 0)	p = 0.392

Allergic bronchopulmonary aspergillosis (ABPA)	3.6% (n = 2)	12.2% (n = 5)	p = 0.229

Pancreatic insufficiency	80.0% (n = 44)	78.1% (n = 32)	p = 0.782

Hepatobiliary complications	23.6% (n = 13)	26.8% (n = 11)	p = 0.840

Distal intestinal obstruction	7.3% (n = 4)	0.0% (n = 0)	p = 0.082

Diabetes mellitus	0.0% (n = 0)	7.3% (n = 3)	p = 0.041

Nasal polyp	10.9% (n = 6)	17.1% (n = 7)	p = 0.366

Isolation obligation for patient	1.8% (n = 1)	9.8% (n = 4)	p = 0.081

Lung function of these patients is worse than normal levels especially for MEF_25_. In addition, the clinical data shows, that 63.6% of all children and 73.2% of the adolescents have a bacterial colonization of their lungs. About 80% of the patients have a pancreatic insufficiency. As expected adolescents (with a longer disease history than children) have a worse health status than those of lower age. A significant difference between age groups can be found especially in patients having a bacterial colonization of the lungs (Mann-Whitney-U-Test, p = 0.011).

### EQ-5D-Y dimensions and visual analogue scale (VAS)

**Figure 1:**
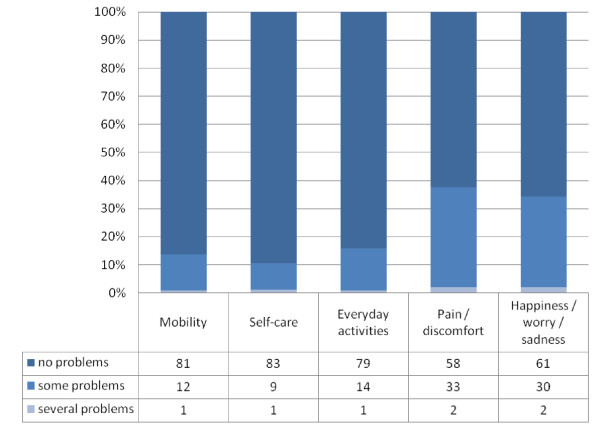
**Distribution of EQ-5D-Y dimensions (n = 94)**.

Figure 1 presents the distribution within the EQ-5D-Y dimensions. Between 84% and 89% of the patients reported no problems (score 1) with 'mobility', 'self-care' and 'usual activities'. 62% to 65% of the patients experienced no problems with 'pain/discomfort' or 'happiness/worry/sadness'. Only one or two patients reported extreme problems (score 3) on at least one EQ-5D-Y dimension.

92 patients had no missing data. Health states were reported from health state 11111 (no problems in all dimensions, n = 41, 44.6%) to health state 22233 (some problems with 'mobility', 'self-care' and 'usual activities' and extreme problems with 'pain/discomfort', n = 1, 1.1%). The high proportion of patients with no problems hints at a ceiling effect typical for a generic instrument like the EQ-5D-Y. However, additional analyses showed significant differences in all but one CFQ subscale ('digestive symptoms, Mann-Whitney-U-Test, p = 0.08) for patients with any problem in comparison to patients without any problems with respect to the EQ-5D-Y. Furthermore, an subgroup analysis for patients who reported no problems on the EQ-5D-Y resulted in no significant differences in all CFQ subscales for patients with different clinical status, for example in pancreatic insufficiency or bacterial colonization of the lungs see table 2.

**Table 2:** Subgroup analysis for patients without problems on EQ-5D-Y

	Pancreatic insufficiency (PI)		Bacterial colonization (BC)	
	
	Mean without PI (SD)/Mean with PI (SD)	Significance level	Mean without BC (SD)/Mean with BC (SD)	Significance level
Physical functioning	91.7 (14.0)/92.2 (12.4)	p = 0.985	95.9 (4.7)/89.4 (15.2)	p = 0.337

Emotional state	85.5 (10.9)/87.5 (10.4)	p = 0.616	88.1 (11.5)/86.1 (9.7)	p = 0.408

Social limitations	66.4 (14.4)/72.6 (11.2)	p = 0.143	73.1 (12.3)/69.7 (11.3)	p = 0.469

Body image	79.4 (17.5)/87.5 (20.0)	p = 0.153	85.2 (24.7)/87.5 (16.2)	p = 0.987

Eating disturbance	88.9 (17.0)/94.8 (9.8)	p = 0.344	91.9 (12.9)/94.7 (10.6)	p = 0.376

Treatment burden	77.8 (17.0)/74.0 (19.3)	p = 0.765	68.1 (20.1)/77.8 (16.8)	p = 0.135

Respiratory symptoms	82.1 (11.7)/86.0 (16.2)	p = 0.212	88.0 (9.3)/83.1 (18.2)	p = 0.509

Digestive symptoms	76.2 (16.3)/78.5 (20.1)	p = 0.613	75.6 (19.3)/80.2 (19.8)	p = 0.457

As a result the EQ-5D-Y showed to be able to discriminate problems in different areas.

90 patients filled out the visual analogue scale about their subjective health perception. The mean VAS for eight to 13-year old patients was 85.4 (SD 16.4), the mean VAS for 14 to 17 year old patients was 79.4 (SD 13.2). Hence, the VAS value is significantly better for younger patients than for older patients (Mann-Whitney-U-Test, p = 0.006).

### Correlations between EQ-5D-Y and CFQ

93 patients completed the disease-specific CFQ. 55 patients between 8 and 13 years as well as their parents reported quality of life by the CFQ-k (for children) and the CFQ-e (for parents). 38 adolescent patients reported their quality of life by the CFQ 14+ (for patients older than 14 years).

Correlations between EQ-5D-Y dimensions and the CFQ scales are reported in table 3. If the correlation (Spearman's rank correlation) is significant at a significance level of 0.01 it is marked with two stars (**). One star (*) indicates that the correlation is significant at a level of 0.05 (non-significant correlations are reported as n.s.).

**Table 3:** Correlations between EQ-5D-Y dimensions and CFQ scales

		Mobility	Self care	Everyday activities	Pain/discomfort	Happiness/worry/sadness	VAS
Physical functioning	CFQk	-0.459 (**)	-0.309 (*)	-0.379 (**)	-0.419 (**)	-0.299 (*)	0.401 (**)
	
	CFQe	-0.516 (**)	-0.307 (*)	-0.438 (**)	-0.417 (**)	-0.267 (n.s.)	0.495 (**)
	
	CFQ 14+	-0.625 (**)	-0.495 (**)	-0.472 (**)	-0.247 (n.s.)	-0.149 (n.s.)	0.576 (**)

Energy	CFQe	-0.248 (n.s.)	-0.181 (n.s.)	-0.263 (n.s.)	-0.509 (**)	-0.315 (*)	0.469 (**)
	
	CFQ 14+	-0.248 (n.s.)	-0.358 (**)	-0.266 (n.s.)	-0.408 (**)	-0.396 (**)	0.726 (**)

Emotional state	CFQk	-0.188 (n.s.)	-0.096 (n.s.)	-0.286 (*)	-0.507 (**)	-0.345 (*)	0.308 (*)
	
	CFQe	-0.428 (**)	-0.317 (*)	-0.402 (**)	-0.517 (**)	-0.421 (**)	0.445 (**)
	
	CFQ 14+	-0.337 (**)	-0.451 (**)	-0.373 (**)	-0.433 (**)	-0.586 (**)	0.567 (**)

Social limitations	CFQk	-0.338 (*)	-0.201 (n.s.)	-0.255 (n.s.)	-0.350 (**)	-0.314 (*)	0.277 (n.s.)
	
	CFQ 14+	-0.255 (n.s.)	-0.200 (n.s.)	-0.309 (n.s.)	-0.315 (n.s.)	0.030 (n.s.)	0.435 (**)

School problems	CFQe	-0.316 (*)	-0.268 (n.s.)	-0.327 (*)	-0.519 (**)	-0.362 (**)	0.452 (**)

Everyday life	CFQ 14+	-0.410 (**)	-0.365 (**)	-0.461 (**)	-0.599 (**)	-0.412 (**)	0.553(**)

Body image	CFQk	-0.171 (n.s.)	-0.178 (n.s.)	-0.292 (*)	-0.293 (*)	-0.391 (**)	0.347 (*)
	
	CFQe	-0.180 (n.s.)	-0.234 (n.s.)	-0.234 (n.s).	-0.279 (*)	-0.265 (n.s.)	0.327 (*)
	
	CFQ 14+	-0.139 (n.s.)	-0.427 (*)	-0.459 (**)	-0.425 (**)	-0.235 (n.s.)	0.551 (**)

Eating disturbance	CFQk	-0.294 (*)	-0.338 (*)	-0.343 (*)	-0.311 (*)	-0.186 (n.s.)	0.187 (n.s.)
	
	CFQe	-0.286 (*)	-0.253 (n.s.)	-0.175 (n.s.)	-0.139 (n.s.)	0.032 (n.s.)	0.170 (n.s.)
	
	CFQ 14+	-0.367 (**)	-0.465 (**)	-0.391 (**)	-0.174 (n.s.)	-0.256 (n.s.)	0.315 (n.s.)

Treatment burden	CFQk	-0.155 (n.s.)	-0.168 (n.s.)	-0.433 (**)	-0.365 (**)	-0.340 (*)	0.166 (n.s.)
	
	CFQe	-0.229 (n.s.)	-0.168 (n.s.)	-0.361 (**)	-0.254 (n.s.)	-0.346 (*)	0.315 (n.s.)
	
	CFQ 14+	-0.298 (n.s.)	-0.473 (**)	-0.282 (n.s.)	-0.217 (n.s.)	-0.320 (n.s.)	0.439 (**)

Subjective health perception	CFQe	-0.336 (*)	-0.308 (*)	-0.380 (**)	-0.508 (**)	-0.364 (**)	0.379 (**)
	
	CFQ 14+	-0.435 (**)	-0.357 (**)	-0.349 (**)	-0.470 (**)	-0.460 (**)	0.744 (**)

Weight problems	CFQe	-0.116 (n.s.)	-0.347 (*)	-0.123 (n.s.)	-0.355 (**)	-0.129 (n.s.)	0.266 (n.s).
	
	CFQ 14+	-0.374 (**)	-0.495 (*)	-0.533 (**)	-0.240 (n.s.)	-0.233 (n.s.)	0.256 (n.s.)

Respiratory symptoms	CFQk	-0.235 (n.s.)	0.004 (n.s.)	-0.328 (*)	-0.315 (*)	-0.304 (*)	0.393 (**)
	
	CFQe	-0.434 (**)	-0.228 (n.s.)	-0.383 (**)	-0.376 (**)	-0.302 (*)	0.450 (**)
	
	CFQ 14+	-0.469 (**)	-0.258 (n.s.)	-0.286 (n.s.)	-0,259 (n.s.)	-0.457 (**)	0.592 (**)

Digestive symptoms	CFQk	-0.157 (n.s.)	-0.309 (*)	-0.380 (**)	-0.474 (**)	-0.066 (n.s.)	0.153 (n.s.)
	
	CFQe	-0.115 (n.s.)	-0.227 (n.s.)	-0.239 (n.s.)	-0.453 (**)	0.056 (n.s.)	0.179 (n.s.)
	
	CFQ 14+	-0.162 (n.s.)	-0.229 (n.s.)	-0.221 (n.s.)	-0.214 (n.s.)	-0.200 (n.s.)	0.496 (**)

CFQk = Cystic Fibrosis Questionaire (version for children from 8 to 13 years)

CFQe = Cystic Fibrosis Questionaire (version for parents of children from 8 to 13 years)

CFQ 14+ = Cystic Fibrosis Questionaire (version for adolescents from 14 to 17 years)

* = significant at p < 0.01

** = significant at p < 0.05

n.s. = not significant

For all CFQ versions the EQ-5D-Y dimension '*mobility' *correlates moderately (0.3 ≤ r_S _≤ 0.5, p < 0.01) or highly (r_S _> 0.5, p < 0.01) with the scale 'physical functioning'. For the dimension '*self care' *several mild (r_S _< 0.3, p < 0.01) or moderate (0.3 ≤ r_S _≤ 0.5, p < 0.01) correlations with CFQ scales can be found, but overall the correlations of this dimension are the lowest in comparison to the other dimensions. The dimension *'everyday activities' *shows several moderate correlations especially with the scales 'physical functioning', 'emotional state', 'everyday life' and 'treatment burden'. Some CFQ scales correlate highly to the EQ-5D-Y dimension '*pain/discomfort'*. For the dimension '*happiness/worry/sadness' *the highest correlation (r_S _= 0.586, p = 0.01) can be found with the CFQ scale 'emotional state' in adolescent patients.

For all CFQ versions the *visual analogue scale *shows moderate or high correlations (r_S _> 0.3, p < 0.01) with the CFQ scales 'physical functioning', 'energy', 'school problems' and 'everyday life', 'subjective health perception', and 'respiratory symptoms'. The overall highest correlation (r_S _= 0.744, p = 0.01) can be found between 'subjective health perception' and the visual analogue scale in adolescent patients.

Only a few statistically significant correlations between clinical data and quality of life data can be found. The dimension 'usual activities' shows low correlations with the '% of vital capacity' (r_S _= 0.248, p < 0.05) and if a patient has to be isolated according to a bacterial colonization of his lungs (r_S _= 0.281, p < 0.01). The visual analogue scale correlates moderately especially with age (r_S _= 0.339, p < 0.01) and weakly with a bacterial colonization of the lungs (r_S _= 0.215, p < 0.05). Therefore differences in clinical data seem not to have a major impact on quality of life data.

## Discussion

### Summary of the results

44.6% of the patients stated that they had no problems in any of the EQ-5D-Y dimensions. The high proportion of patients with no problems hints at a ceiling effect typical for a generic instrument like the EQ-5D-Y. However, additional analyses showed that the EQ-5D-Y is able to discriminate problems in different areas.

Several low to strong correlations between the dimensions of the EQ-5D-Y and the scales of the CFQ for children, their parents and adolescents could be found in the analysis. Looking at the five EQ-5D-Y dimensions the highest correlation (r_S _= 0.625, p = 0.01) was found between the dimension 'mobility' and the CFQ scale 'physical functioning' in adolescent patients. For all CFQ versions the *visual analogue scale *showed moderate or high correlations (r_S _> 0.3) with several CFQ scales. The overall highest correlation was found between the 'subjective health perception' and the visual analogue scale (r_S _= 0.744, p = 0.01) in adolescents. Hence, the hypothesized associations were confirmed.

Only a few correlations between clinical data and quality of life data were found. Therefore, differences in clinical data may not have a major impact on quality of life data for cystic fibrosis children and adolescents.

### Interpretation of the results

It is noticeable that in many cases patients reported about a good EQ-5D health state despite their strong disease according to the medical classification of disease activity. The objective health state may be actually lower (worse) than reported. An explanation might be coping, which means that patients learn to live with the disease and do not perceive limitations as bad as expected.

Different significance levels for correlations between EQ-5D-Y dimensions and CFQ scales might result from the fact, that some dimensions of the EQ-5D-Y and scales of the CFQ are directed at exactly the same issue (for example the visual analogue scale of the EQ-5D-Y and the CFQ scale 'subjective health perception') and others only partially. Furthermore a reason for higher correlations of the visual analogue scale in comparison to the EQ-5D-Y dimensions might be the continuous character of the scale instead of the three score levels.

Although the time frame of the EQ-5D-Y (today) is different to the time frame of the CFQ (last two weeks), significant correlation results were found. However, it is possible that correlations within the same time frame still would be higher than those reported.

For further research it would be interesting to compare different properties of the used measures in a setting with multiple and continuous assessments at various time points.

## Conclusion

Data was collected in a multi centre study from a representative patient group of children and adolescents with cystic fibrosis. The analysis showed several correlations between the different dimensions and the visual analogue scale of the EQ-5D-Y and the scales of the different CFQ versions for children, their parents and adolescents. Therefore, the EQ-5D-Y can be considered a cross-sectional valid generic health outcome measure which reflects differences in health according to the progression of the life long chronic disease cystic fibrosis.

## Competing interests

The authors declare that they have no competing interests.

## Authors' contributions

DE-K collected the data, did the statistical analyses and writing of the manuscript. TM and WG participated in conception, design, and interpretation of data as well as revision of the manuscript. All authors read and approved the final manuscript.

## Pre-publication history

The pre-publication history for this paper can be accessed here:


